# The Surgical Significance of Vomiting.—II

**Published:** 1908-05-16

**Authors:** 


### Points in Surgery.

### THE SURGICAL SIGNIFICANCE OF VOMITING.

### II.—POST-OPERATIVE VOMITING.

Vomiting occurring after an operation may or may not be due to the anæsthetic. It is at any rate a common habit among surgeons to attribute post-operative vomiting to this cause, an accusation which anæsthetists are accustomed to repudiate with corresponding vigour. It is as erroneous to suppose that all cases of post-operative vomiting are due to the anæsthetic as it is to deny that vomiting ever occurs as the result of these drugs. It is, for instance, the rule rather than the exception to have some vomiting after the administration of ether; after chloroform it is more rare, but when it does occur it is generally more serious and persistent. It has been suggested that the ether is largely excreted by the gastric mucus membrane, collects in the stomach combined with mucus, and thus excites vomiting either by acting as a direct irritant or by setting up a gastritis. The fact that the ether found in the stomach must have been excreted by that organ is not, however, very easy to establish, since some of the drug is swallowed at the time of operation. Post-anæsthetic vomiting is usually easy to deal with; a simple and efficient plan is to wash out the stomach with a warm solution of sodium bicarbonate. The bicarbonate frees the mucus, and so allows the stomach contents to come away quite easily; or simply to give warm water, about half a pint, to drink may do as well, because it will cause the patient to vomit and so empty the stomach. Subsequently cocaine gr. $\frac{1}{20}$ may be given, which blunts the sensibility of the nerve-endings in the gastric mucous membrane, and so prevents the irritating effect of any ether which may still be retained or may subsequently be excreted. Many other drugs have been tried, such as two minims of tincture of iodine or two or three minims of dilute hydrocyanic acid; but their action is not reliable, and Mummery, in his "After-treatment of Operations," advises that "in cases of severe and intractable vomiting the stomach should be washed out with warm water by means of a soft rubber tube, until the water comes back quite clear; this removes any decomposing or irritating material present." The writer is able to endorse these remarks from personal experience, and can recall not a few cases where this put an end to the sickness when all other methods had failed.

Apart from these there are, however, cases in which the vomiting must be regarded as an effect of the operation and not of the anæsthetic. These cases are met with almost exclusively after operations upon the abdomen, as might be expected. In gastro-jejunostomy, for instance, vomiting may be a most troublesome complication. It is due, when it occurs, to faulty technique in performing the operation, so that what is known as a vicious circle is set up. That is to say that the contents of the stomach, after passing through the artificial opening, travel into the proximal loop of the small intestine instead of into the distal one, as they should do, and so cause a regurgitation of bile from the duodenum into the stomach and set up emesis. This complication was more frequently met with in the early days of gastro-jejunostomy than it is now, when experience has improved the technique of the operation. The most difficult detail in performing a gastro-jejunostomy is the selection of a portion of small intestine at the right distance from the duodeno-jejunal flexure to perform the anastomosis. If the distance is too short there will be tension on the stitches, and they will tear through, but if, on the other hand, it is too long, the proximal loop will sag down and the complication already referred to will ensue. Many plans have been invented to deal with a vicious circle after gastro-jejunostomy. It may be said with confidence that those which depend on the formation of a valve of mucous membrane at the anastomosis are doomed to failure. Perhaps the simplest plan is to open the abdomen again and to perform a lateral anastomosis between the coils of small intestine which lead respectively towards and away from the gastro-jejunostomy. Any bowel contents which may collect in the duodenum or proximal loop can then make their way onwards through this second opening. So great was the terror of a vicious circle following a gastro-jejunostomy in the early days of the operation that an ingenious alternative method of performing the operation was devised for the purpose of avoiding this. The intestine was divided transversely a few inches from the duodeno-jejunal flexure. The distal end was implanted into the stomach and the proximal cut end was implanted into the small intestine at some distance below the union with the stomach. The method was tried in a number of cases, and although it was successful as far as it went, it was found to be a laborious proceeding and has fallen into disuse. Now that we have had a chance of examining post-mortem the bodies of patients who have lived some time after a gastro-jejunostomy, we know that, in whatever part of the stomach the opening was originally made, that part eventually becomes dragged down, and forms, as it were, the apex of a funnel, and that when this occurs vomiting is likely to cease.

Vomiting is also a prominent symptom after operation in cases of general peritonitis, and in association with difficulty or failure in getting the bowels to act, must be taken as an indication that there is commencing or actual paralysis of the intestinal wall, a condition which calls for the adoption of immediate and drastic measures, as has been recently explained in an article on "Ileus following General Peritonitis" in these columns. Vomiting may occur also after simple operations on the stomach, and is best explained by supposing that the mechanical interference with the organ has disturbed its function, or that dragging on the sympathetic plexus has produced this effect. The persistence of vomiting after an apparently successful operation for obstructed or strangulated hernia should arouse suspicion. It may betoken a reduction *en masse* or the onset of gangrene of the gut.

